# Epidemiology and Characterization of CTX-M-55-Type Extended-Spectrum β-Lactamase-Producing *Salmonella enterica* Serovar Enteritidis Isolated from Patients in Shanghai, China

**DOI:** 10.3390/microorganisms9020260

**Published:** 2021-01-27

**Authors:** Chenyang Cao, Qinya Niu, Jia Chen, Xuebin Xu, Huanjing Sheng, Shenghui Cui, Bin Liu, Baowei Yang

**Affiliations:** 1 College of Food Science and Engineering, Northwest A&F University, Yangling 712100, China; chenyangcao@nwafu.edu.cn (C.C.); nqy736461094@nwafu.edn.cn (Q.N.); shenghuanjing@nwafu.edu.cn (H.S.); liubin7723@nwsuaf.edu.cn (B.L.); 2 College of Chemical Technology, Shijiazhuang University, Shijiazhuang 050035, China; chenjia0311@aliyun.com; 3 Shanghai Municipal Center for Disease Control & Prevention, Shanghai 200336, China; xuxuebin@scdc.sh.cn; 4 National Institutes for Food and Drug Control, Beijing 100050, China; cuishenghui@aliyun.com

**Keywords:** *Salmonella* Enteritidis, human patients, extended-spectrum β-lactamases, *bla*_CTX-M-55_, pulsed-field gel electrophoresis

## Abstract

The emergence of extended-spectrum β-lactamase-producing *Salmonella enterica* serovar Enteritidis (ESBL-SE) in humans and foods has gained global attention. In particular, CTX-M-type ESBL-SE are increasingly being detected from various sample types. The aim of this study was to comprehensively analyze the epidemiology and characteristics of *bla*_CTX-M-55_-carrying ESBL-SE isolates of clinical origin in Shanghai, China. A total of 292 *S.* Enteritidis isolates were recovered from the feces and blood of outpatients and inpatients between 2006 and 2014. Overall, there was a high frequency of cefotaxime-resistant isolates (97.3%), which was significantly higher (*p* < 0.01) than that of isolates resistant to the other tested antibiotics. All *S.* Enteritidis isolates exhibited resistance to ≥1 antibiotic, and 98.0% were multidrug resistant. A total of 233 isolates were identified as ESBL-SE, 166 of which were CTX-M type. Six subtypes of CTX-M-encoding genes were detected, among which *bla*_CTX-M-55_ (91.6%, 152/166) was the most prevalent genotype. There was high genetic similarity among *bla*_CTX-M-55_-positive ESBL-SE. The *bla*_CTX-M-55_ gene in the ESBL-SE donor strains could be easily transferred into *Enterobacteriaceae* recipient strains. This study highlights that CTX-M-55 should be considered an important surveillance target in Shanghai, China. Cephalosporins, especially cefotaxime, must be used with caution in empirical treatment for *Salmonella* infections.

## 1. Introduction

*Salmonella* is one of the most common pathogens in humans and animals, and *Salmonella* infection imposes a heavy burden on global public health. Gastroenteritis caused by *Salmonella* (salmonellosis) always presents with clinical symptoms (e.g., headache, abdominal pain, and diarrhea) [1,2]. It is estimated that salmonellosis occurs worldwide with more than 93.8 million cases each year, leading to 155,000 deaths [3]. Thus far, more than 2610 *Salmonella* serovars have been identified, while salmonellosis is caused mainly by *S. enterica* serovars Typhimurium and Enteritidis [4]. During the 1990s, *S*. Enteritidis replaced *S*. Typhimurium as the most prevalent *Salmonella* serovar worldwide [5,6]. Although *S.* Enteritidis usually causes predominantly self-limiting enteric disease, some severe clinical illnesses require antibiotics. In particular, fluoroquinolones and extended-spectrum cephalosporins, such as cefotaxime and ceftazidime, are considered first-line clinical drugs [7,8].

In recent decades, the overall antibiotic resistance rate among *Salmonella* has increased dramatically, from 20–30% in the 1990s to 70% in the early 21st century [9]. Abuse and inappropriate usage of antibiotics has caused the spread of multidrug resistance (MDR) among pathogen groups [10,11]. Extended-spectrum cephalosporins are widely used in clinical treatment because of their excellent therapeutic effects. Members of *Enterobacteriaceae*, especially *Escherichia coli* and *Klebsiella pneumoniae*, are common causes of resistance to the third- and fourth-generation cephalosporins, which is often ascribed to the production of extended-spectrum β-lactamases (ESBLs) [12,13]. Moreover, ESBL-producing strains of *Salmonella* have been reported in many regions throughout China, including Beijing, Shanghai, Guangdong, and Shandong [14,15,16,17]. Worse, ESBL-producing *S.* Enteritidis (ESBL-SE) have increasingly been detected from human patients, food animals, and even environmental water [18,19,20,21], which greatly limits the curative effects of antibiotics and increases morbidity and mortality.

The predominant types of ESBLs in *Enterobacteriaceae* are TEM, SHV, and CTX-M, while the most commonly encountered ESBL-encoding gene during the last decade was *bla*_CTX-M_ [17,22]. *bla*_CTX-M_ genes are commonly carried by highly mobile genetic elements (e.g., plasmids and transposons), which might also contain other antibiotic resistance genes [23]. ESBL-SE are commonly considered donors for the transmission of antibiotic resistance through mobile genetic elements among different hosts [20]. Worryingly, CTX-M-type ESBL-SE are increasingly being detected from various sample types in multiple countries, such as China, the United States, England, Wales, and Japan [24]. As *bla*_CTX-M-55_-positive ESBL-SE show high-level resistance to extended-spectrum cephalosporins and often cross-tolerate other antibiotics [15], more attention should be focused on the prevalence of *bla*_CTX-M-55_-carrying ESBL-SE.

To date, studies associated with CTX-M-type ESBL-producing *E.* *coli* and *K. pneumonia* have been conducted around the world [14,25,26]. However, comprehensive studies on the epidemiology and characteristics of *bla*_CTX-M-55_-positive ESBL-SE isolates recovered from human patients are limited. In the present study, we conducted a systematic analysis of the epidemiology of ESBL-SE isolates and the characteristics of *bla*_CTX-M-55_-carrying ESBL-SE isolates recovered from human patients in Shanghai, China, from 2006 to 2014.

## 2. Materials and Methods

### 2.1. Bacterial Isolates 

**Figure 1:**
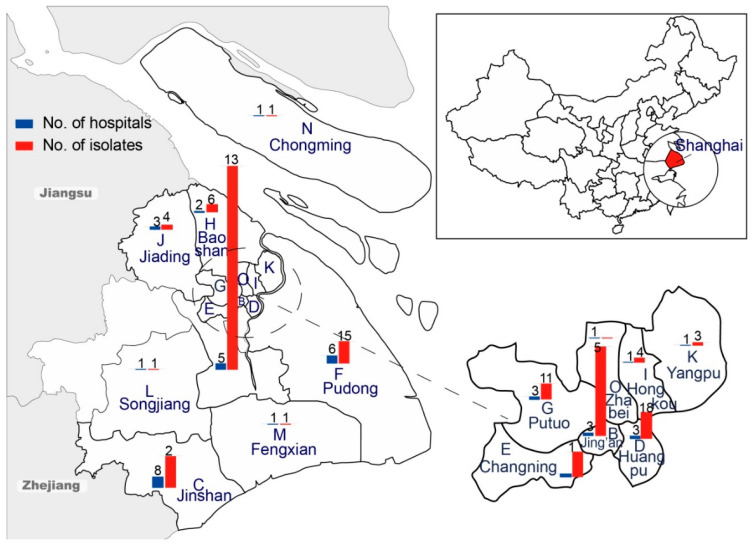
Origins of 292 *S. enterica* serovar Enteritidis isolates from 42 hospitals in 15 districts in Shanghai, China (2006–2014).

A total of 292 *S*. Enteritidis strains were recovered from the feces (*n* = 291) and blood (*n* = 1) of outpatients and inpatients at 42 hospitals in 15 districts in Shanghai, China, between 2006 and 2014 (Figure 1). The strains were provided by the Municipal Center for Disease Control & Prevention (Shanghai, China). Serotyping was performed according to the Kauffmann-White scheme and the manufacturer’s instructions via slide agglutination, using *Salmonella*-specific hyperimmune sera (S&A Reagents Lab Ltd., Bangkok, Thailand). The *Salmonella* strains were maintained in Luria-Bertani broth (Beijing Land Bridge Technology Co., Ltd., Beijing, China) containing 40% glycerol and stored at −80 °C until use.

### 2.2. Antibiotic Susceptibility Testing 

The antibiotic susceptibility of the isolates was determined using the Kirby-Bauer disk diffusion method according to the guidelines recommended by the Clinical and Laboratory Standard Institute (CLSI) [27]. The test was performed on Mueller-Hinton agar (Beijing Land Bridge Technology Co., Ltd., Beijing, China) with the following 14 antibiotic disks (Oxoid, Basingstoke, UK): 30 μg cefotaxime, 30 μg ceftazidime, 30 μg tetracycline, 20/10 μg amoxicillin/clavulanic acid, 10 μg ampicillin, 23.75/1.25 μg sulfamethoxazole/trimethoprim, 5 μg ciprofloxacin, 30 μg chloramphenicol, 5 μg ofloxacin, 30 μg nalidixic acid, 5 μg trimethoprim, 10 μg gentamicin, 300 μg sulfisoxazole, and 10 μg streptomycin. The minimum inhibitory concentrations (MICs) of selected cephalosporins for ESBL-SE isolates were determined by agar dilution using Mueller-Hinton agar in accordance with CLSI standards and guidelines. The following nine cephalosporins (Sigma Aldrich, St. Louis, MO, USA) were tested: cefotaxime, ceftazidime, ceftriaxone, cefepime, cefazolin, cefpodoxime, cefoxitin, cefotaxime-clavulanic acid, and ceftazidime-clavulanic acid. *E. coli* ATCC25922 and *Enterococcus faecalis* ATCC29212 were used as quality control organisms in the antibiotic MIC assays; the results were interpreted according to the 2015 CLSI guidelines.

### 2.3. Detection of ESBL Phenotypes and Sequencing of ESBL-Encoding Genes 

ESBL-SE isolates were screened and confirmed by double-disk synergy testing as previously described [28]. Antibiotic disks of 30/10 μg cefotaxime/clavulanic acid and 30/10 μg ceftazidime/clavulanic acid were obtained from Becton Dickinson (Franklin Lakes, NJ, USA); disks of 30 μg cefotaxime and 30 μg ceftazidime were obtained from Oxoid. The phenotypic expression of ESBLs was interpreted according to the 2015 CLSI guidelines.

Further, all ESBL-SE isolates underwent PCR detection for the presence of ESBL-encoding genes (i.e., *bla*_CTX-M_, *bla*_TEM_, *bla*_OXA_, *bla*_CMY_, *bla*_PSE_, *bla*_PER_, *bla*_IMP_, *bla*_VIM_, *bla*_ACC_, *bla*_GES_) as previously described [21,29,30,31]. The nucleotide sequences of PCR primers are shown in Table S1. All PCR products of ESBL-encoding genes were sequenced at AuGCT Biotechnology Co., Ltd. (Beijing, China). The DNA sequence data were checked for ESBL gene subtypes using BLAST (http://www.ncbi.nlm.nih.gov/BLAST/).

### 2.4. Pulsed-Field Gel Electrophoresis (PFGE)

All *bla*_CTX-M-55_-positive ESBL-SE isolates were subtyped by PFGE following digestion with *Xba*I (TaKaRa, Dalian, China) according to the Centers for Disease Control and Prevention (CDC) protocols [32]. The digested DNA fragments were separated by a CHEF Mapper electrophoresis system (Bio-Rad Laboratories, Hercules, CA, USA) as previously described [33]. *S. enterica* serovar Braenderup H9812 was used as the standard control. The PFGE patterns were interpreted with the BioNumerics software (version 3.0; Applied Maths, Sint-Martens-Latem, Belgium). The similarity index of the DNA profiles of the isolates was calculated using the Different ban correlation coefficient option of the software with a position tolerance of 0.9% and an optimization of 1.5%. The unweighted-pair group method using average linkages (UPGMA) was used to construct a dendrogram. Profiles were considered to be different if they differed by one band.

### 2.5. Conjugation Experiment

Conjugation was performed using a previously described method [34]. Three representative *bla*_CTX-M-55_-positive ESBL-SE isolates were selected as donors. The recipients were rifampin-resistant and ceftazidime-susceptible *S.* Enteritidis isolate (isolate 20, from this study) and *E. coli* C600 (from our laboratory), and one ciprofloxacin-resistant and cefotaxime-susceptible foodborne *E. coli* isolate (strain 1-22, from our laboratory; Table S2). Transconjugants were selected on Luria-Bertani agar (Beijing Land Bridge Technology Co., Ltd.) containing two different antibiotics. Antibiotic susceptibility and presence of the *bla*_CTX-M-55_ gene in the transconjugants were determined as described above.

### 2.6. Statistical Analysis

As we recovered limited numbers of *S.* Enteritidis isolates in the first five years, the isolates obtained in 2006–2010 were combined to perform statistical analysis. The relationship between the detection rate of antibiotic-resistant isolates and the sampling year, district, and patient gender and age were examined by redundancy analysis using Canoco 4.5 (Microcomputer Power, Ithaca, NY, USA). The detection rates of antibiotic-resistant isolates, ESBL-SE isolates, and the *bla*_CTX-M_ gene were compared among the investigated groups using GraphPad Prism 7.0 (GraphPad Software, La Jolla, CA, USA) and SPSS 20.0 (IBM SPSS, Armonk, NY, USA). The *χ*^2^ test was used to assess whether the difference detected in the rates of specific isolates was statistically significant (*p* < 0.05). Odds ratios (OR) with 95% confidence intervals (95% CI) for ESBL-SE were estimated using logistic regression in SPSS 20.0, unadjusted for year, source, district, and hospital.

## 3. Results

### 3.1. Antibiotic Susceptibility

Of the 292 *S.* Enteritidis isolates, 284 were resistant to cefotaxime. The detection rate of cefotaxime-resistant isolates (97.3%) was significantly higher (*p* < 0.01) than that of isolates resistant to any of the other 13 tested antibiotics. The detection rate (3.8%) of ofloxacin-resistant isolates was significantly lower (*p* < 0.01) than that of isolates resisted to any of the other 13 antibiotics. All *S*. Enteritidis isolates were resistant to at least one antibiotic tested, while 112 (38.4%) were co-resistant to 2–4 antibiotics, 85 (29.1%) to 5–7 antibiotics, 84 (28.8%) to 8–10 antibiotics, and 10 (3.4%) to >10 antibiotics.

**Figure 2:**
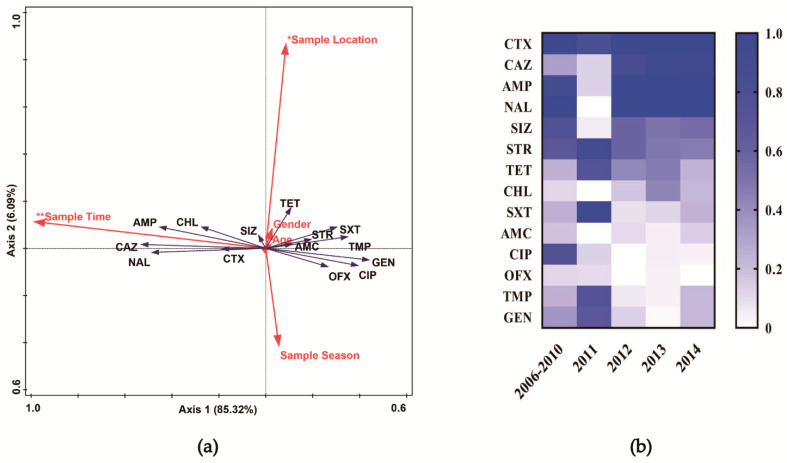
(**a**) Biplot of the redundancy analysis depicting the relationship between antibiotic resistance rates of *S.* Enteritidis isolates and sampling time, location, season, and patient gender and age (*n* = 292). * *p* < 0.05; ** *p* < 0.01 (by Mantel test). (**b**) Antibiotic resistance rates of *S.* Enteritidis isolates recovered from clinical samples in Shanghai, 2006–2014 (*n* = 292). CTX: Cefotaxime; CAZ: Ceftazidime; TET: Tetracycline; AMC: Amoxicillin-Clavulanic acid; AMP: Ampicillin; CIP: Ciprofloxacin; SXT: Sulfamethoxazole/Trimethoprim; CHL: Chloramphenicol; OFX: Ofloxacin; NAL: Nalidixic acid; TMP: Trimethoprim; GEN: Gentamicin; SIZ: Sulfisoxazole; STR: Streptomycin.

The results of redundancy analysis (Figure 2a) indicated that the distribution of the antibiotic-resistant isolates was significantly influenced by sampling time (*p* < 0.01). Sampling district also had a significant influence (*p* < 0.05) on the distribution of antibiotic resistance among the isolates. There was no significant relationship between the distribution of antibiotic resistance and the sampling season, patient gender, and patient age.

Many of the isolates recovered in 2006–2010, 2011, 2012, 2013, and 2014 were resistant to multiple antibiotics, and the rates of resistant isolates significantly changed (*p* < 0.05) among the 14 antibiotics tested (Table S3). Except for 2011, all (100.0%) isolates were resistant to cefotaxime and the detection rate was the highest (*p* < 0.05) compared with that to other antibiotics in different years (Figure 2b, Table S3).

### 3.2. Characterization of ESBL-SE

Of the 292 *S*. Enteritidis isolates, 233 (79.8%) were ESBL positive. The detection rate of ESBL-SE showed an increasing tendency from 2006 to 2012 and then decreased slightly in 2013–2014 (Figure S1). The rates of ESBL-SE detected in 2012 (95.8%, 46/48), 2013 (94.2%, 131/139), and 2014 (83.8%, 31/37) were significantly higher (*p* < 0.01) than that in 2006–2011 (21.4–47.5%; Figure S1). Logistic analyses revealed that the distribution of ESBL-SE isolates changed significantly over the years. Compared with Minhang district, ESBL-SE were more likely to be detected in the Huangpu (unadjusted OR, 0.2; 95% CI, 0.06–0.5) and Changning districts (unadjusted OR, 0.2; 95% CI, 0.08–0.7). In addition, ESBL-SE appeared to be more prevalent in pediatric hospitals than in public hospitals (unadjusted OR, 2.1; 95% CI, 1.2–3.9; Table 1).

**Figure 3:**
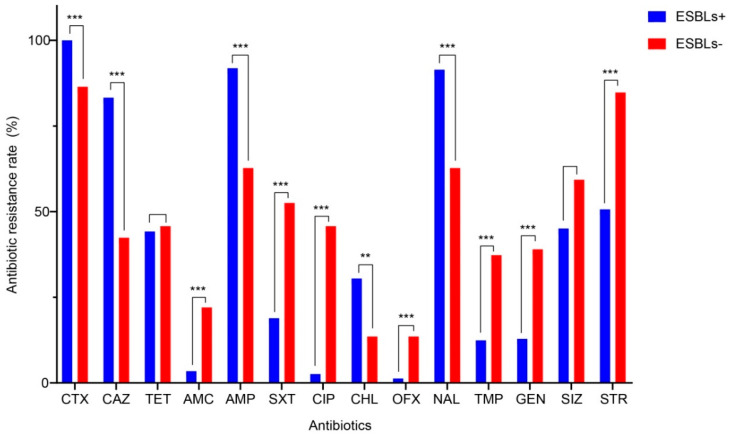
Comparison of multidrug resistance rates between ESBL-producing (ESBLs^+^, *n* = 233) and non-ESBL-producing (ESBLs^−^, *n* = 59) *S.* Enteritidis isolates. ** *p* < 0.01; *** *p* < 0.001 (by *χ*^2^ test). CTX: Cefotaxime; CAZ: Ceftazidime; TET: Tetracycline; AMC: Amoxicillin-Clavulanic acid; AMP: Ampicillin; SXT: Sulfamethoxazole/Trimethoprim; CIP: Ciprofloxacin; CHL: Chloramphenicol; OFX: Ofloxacin; NAL: Nalidixic acid; TMP: Trimethoprim; GEN: Gentamicin; SIZ: Sulfisoxazole; STR: Streptomycin.

**Figure 4:**
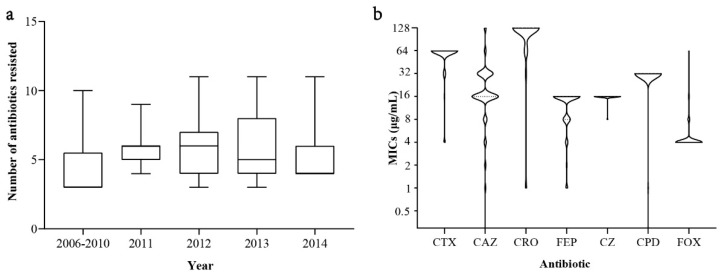
The total number of antibiotics to which the Salmonella isolates were resistant (**a**) and the minimum inhibitory concentrations (MICs; **b**) of cephalosporins for ESBL-producing Enteritidis isolates recovered from different years. CTX: Cefotaxime; CAZ: Ceftazidime; CRO: Ceftriaxone; FEP: Cefepime; CZ: Cefazolin; CPD: Cefpodoxime; FOX: Cefoxitin.

Compared with ESBL-negative SE isolates, MDR ESBL-SE isolates displayed significantly higher (*p* < 0.01) resistance rates to cefotaxime, ceftazidime, ampicillin, chloramphenicol, and nalidixic acid. No significant difference was found between the detection rates of ESBL-SE and non-E-SBL-SE isolates resistant to tetracycline or sulfisoxazole (Figure 3). Considering the total number of antibiotics to which the *Salmonella* isolates were co-resistant (Figure 4a), the ESBL-SE isolates recovered from 2011 were co-resistant to more antibiotics compared with those from the other years (median, 6 vs. 5; *p* < 0.01). No significant difference was found in the MDR patterns of ESBL-SE over the period 2006–2014.

The ESBL-SE isolates were also tolerant of some cephalosporins at high concentrations. All ESBL-SE isolates (233, 100.0%) were resistant to cefotaxime, and 224 (96.1%) were resistant to ceftriaxone, cefazolin, and cefpodoxime. The most common MICs of these four antibiotics for the ESBL-SE isolates were 64 μg/mL (16-fold to the breakpoint), 128 μg/mL (32-fold to the breakpoint), 16 μg/mL (2-fold to the breakpoint), and 32 μg/mL (4-fold to the breakpoint), respectively (Figure 4b, Table S4). However, clavulanic acid partially or completely restored cefotaxime and ceftazidime activity; their MICs were mostly 0.12 μg/mL and 0.5 μg/mL, respectively (data not shown).

### 3.3. Prevalence of ESBL-Encoding Genes and Description of bla_CTX-M-55_-Positive ESBL-SE

Ten ESBL-encoding genes were screened in the 233 ESBL-SE isolates by PCR and confirmed by DNA sequencing. Among these, 71.2% and 25.3% of the isolates harbored *bla*_CTX-M_ and *bla*_TEM_, respectively. The *bla*_CTX-M_ gene sequences revealed six subtypes, *bla*_CTX-M-55_ (91.6%, 152/166), *bla*_CTX-M-64_ (2.4%, 4/166), *bla*_CTX-M-123_ (2.4%, 4/166), *bla*_CTX-M-3_ (1.8%, 3/166), *bla*_CTX-M-79_ (1.2%, 2/166), and *bla*_CTX-M-15_ (0.6%, 1/166). Approximately 54.5% and 21.0% of the ESBL-SE isolates carried one and two ESBL-encoding genes, respectively. The most prevalent genes coexisting in the isolates were *bla*_CTX-M-55_/*bla*_TEM-1_ (77.6%, 38/49).

Although *bla*_CTX-M-55_ was not detected in 2006–2010, its prevalence was significantly increased (*p* < 0.01) in 2011 (68.4%), and was maintained at high levels in the following three years (60.9–67.7%; Table 2). The *bla*_CTX-M-55_-positive ESBL-SE isolates could tolerate higher concentrations of cefotaxime than the *bla*_CTX-M-55_-negative isolates. All *bla*_CTX-M-55_-positive isolates were resistant to cefotaxime, and 149 (98.0%) were resistant to ceftriaxone, cefazolin, and cefpodoxime. When compared with the *bla*_CTX-M-55_-negative isolates, the *bla*_CTX-M-55_-positive isolates were significantly more frequently resistant (*p* < 0.01) to third-generation cephalosporin (ceftazidime) and fourth-generation cephalosporin (cefepime; Table 3).

### 3.4. PFGE Patterns of bla_CTX-M-55_-Positive ESBL-SE

**Figure 5:**
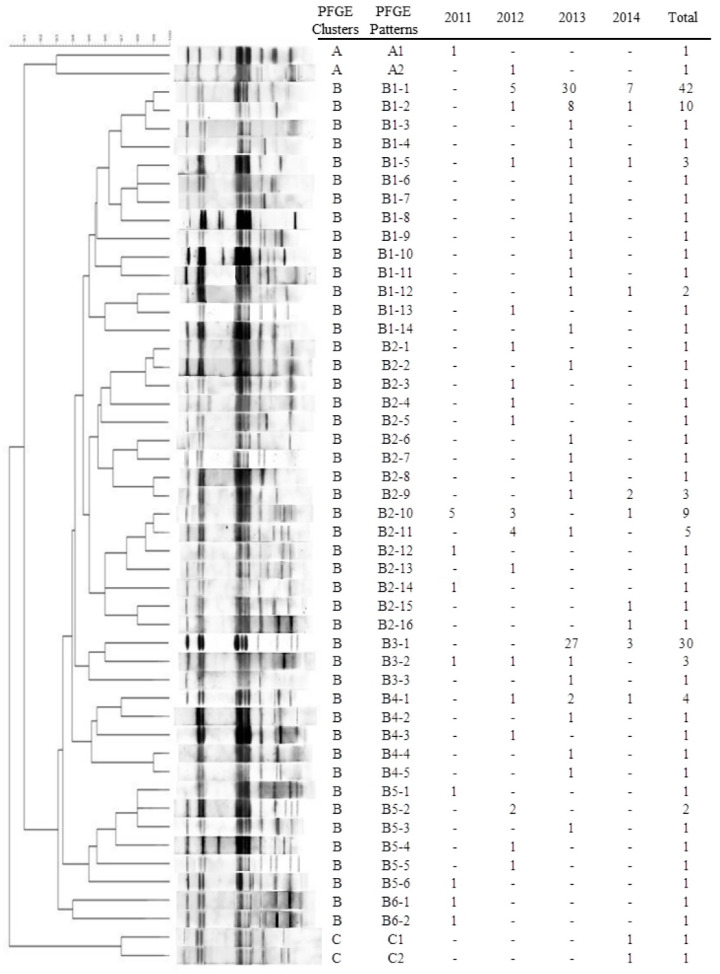
Dendrogram of patterns generated by pulsed-field gel electrophoresis (PFGE) of 152 *bla*_CTX-M-55_-postitive *S.* Enteritidis isolates. *Xba*I was used as the PFGE restriction enzyme. The data in columns 2011, 2012, 2013, and 2014 are the number of isolates from which each pattern was recovered.

Fifty distinct PFGE patterns were identified from 152 *bla*_CTX-M-55_-positive ESBL-SE isolates (Figure 5). PFGE genotypes were grouped into three clusters (A, B, C). Cluster A had 2 patterns; cluster B and C had 46 and two patterns, respectively. The isolates recovered in 2011 had nine patterns, while the isolates recovered in 2012, 2013, and 2014 had 18, 27, and 12 patterns, respectively. Four patterns in the isolates lasted for 2 years, and six patterns lasted for 3 years.

Among the three clusters, cluster B contained *bla*_CTX-M-55_-postitive 148 isolates, 42 of which had the same PFGE pattern (type B1-1). Similar results were found for the isolates of types B3-1, B1-2, and B2-10. Although several *bla*_CTX-M-55_-postitive isolates were recovered from different years, districts, hospitals, and outpatients, they nevertheless shared identical DNA profiles (Table S5). In type B4-1, the PFGE pattern of one isolate recovered from blood (isolate 114) was identical to that of three isolates recovered from fecal samples (isolate 273, 244, 225).

### 3.5. Conjugation of Three bla_CTX-M-55_-Postitive ESBL-SE

Based on their PFGE pattern, antibiotic resistance profile, ESBL-encoding gene, and year of isolation, three *bla*_CTX-M-55_-postitive *S.* Enteritidis isolates (isolate 71, 86, 122) were selected as donors for conjugation. The results showed that filter-mating could successfully transfer *bla*_CTX-M-55_ and the antibiotic resistance phenotype of the donor. The cefotaxime- and ceftazidime-susceptible recipients exhibited partial or complete cefotaxime and ceftazidime resistance phenotypes after conjugation with the three donors (Table S6). Other resistance phenotypes, such as resistance to ampicillin, sulfamethoxazole/trimethoprim, and chloramphenicol, were also co-transferred from specific donor strains to *E. coli* C600. *bla*_CTX-M-55_ and *bla*_CTX-M-55_/*bla*_TEM-1_ from the donors could all be detected in the corresponding transconjugants. The three donors transferred their ESBL-encoding gene to *S.* Enteritidis 20 at rates ranging from 4.6 × 10^−2^ to 6.2 × 10^−1^ transconjugants per recipient cell, and to *E. coli* C600 at 1.5 × 10^−2^ to 4.2 × 10^−2^ transconjugants per recipient cell (Table S6).

## 4. Discussion

*Salmonella* infection has a huge impact on global human health [35]. Excessive consumption of extended-spectrum β-lactam antibiotics has potentially promoted the spread of ESBL-SE [28]. Thus far, more than 200 CTX-M lactamase variants have been reported, based on their variable substrate affinities. Based on amino acid sequence homology, these CTX-M lactamases can be clustered into five subgroups: CTX-M-1, CTX-M-2, CTX-M-8, CTX-M-9, and CTX-M-25. CTX-M-55, a derivate of CTX-M-15, also belongs to the CTX-M-1 group [17,36]. However, there is limited information on *bla*_CTX-M-55_-carrying ESBL-SE in human patients worldwide. In the present study, we surveyed the antibiotic resistance profiles of *S.* Enteritidis, the prevalence of ESBL-SE, and demonstrated the presence and transmission of ESBL-encoding genes (especially *bla*_CTX-M-55_) in ESBL-SE isolates from human patients in Shanghai, China.

We found that the 292 *Salmonella* strains showed significant variation in antibiotic resistance rate from 2006 to 2014 (Table S3). This finding is consistent with a previous report in the same region (Shanghai, China), where *S.* Enteritidis isolates exhibited an increasing frequency of resistance to cephalosporins from 2006 to 2012 [37]. This is especially notable given that almost all isolates tested in the present study were resistant to cefotaxime, a third-generation cephalosporin (Figure 2b). According to the People’s Republic of China National Health Commission report, third-generation cephalosporins are widely used in general hospitals, with the top three being ceftazidime, ceftriaxone, and cefmenoxime in 2018 [38]. Although there was insufficient information on the use of cephalosporins in Chinese hospitals during 2006–2014, it is possible that the high prevalence of cefotaxime resistance was caused by the overuse of cephalosporins.

Through the redundancy analysis, we explored the relationship between the antibiotic resistance of *S.* Enteritidis isolates and their sampling time, location, and patient age and gender, we found that the antibiotic resistance rate was considerably influenced by sampling time and location, especially for ceftazidime, gentamicin, ampicillin, nalidixic acid, and chloramphenicol (Figure 2a). According to these data, we can infer that antibiotic resistance is a common phenomenon among *S.* Enteritidis in human patients in Shanghai. As *S.* Enteritidis resistance to ceftazidime has risen in Shanghai over the investigated period, third line should be avoided as empirical treatment of salmonellosis.

The problems of resistant *Salmonella* highlight the urgent need for new antibiotics to treat infections caused by them. However, the process of manufacturing new antibiotic usually takes a very long time to be commercially ready. Therefore, it is very crucial to search for an effective alternative replacing the use of synthetic compounds. Recently, many researchers have reported that transporter inhibitors show strengthen antibiotic effect against resistant strains [39,40,41]. Essential oils extracted from plants and spices also exhibited the bactericidal potential against pathogens [42,43,44]. The development of alternative synthetic compounds might contribute to discover novel therapeutic approaches for tackling infections.

Of the 292 *S*. Enteritidis isolates tested in this study, 79.8% produced ESBLs, while 96.1% of the ESBL-SE isolates exhibited resistance to >3 antibiotics (Figure 4a). The detection rate of ESBL-SE of clinical origin in Shanghai is higher than the previously reported 60.9% in patients and foods in the same region [19,20]. This discrepancy may be caused by the difference in the studied population. To assess the association between ESBL-SE and clinical characteristics, we compared the unadjusted ORs of ESBL-SE and non–ESBL-SE isolates. Logistic regression showed that year of isolation, outpatient and hospital type of patient visit, and administrative district were all associated with the presence of ESBL-SE (Table 1). Therefore, there should be more focus on ESBL-SE infections in children in Shanghai, as they cause large-scale morbidity and mortality. Our results are in accord with recent studies indicating that more attention should be paid to *S.* Enteritidis infections in young children in China [19,45].

Our data indicated that not only was there an alarming increase in the detection rate for ESBL-SE strains over time, but there was also significant resistance to multiple antibiotics among the ESBL-SE (Figure 3). A recent study on ESBL-producing *Salmonella* from Cambodian retail meats also reported overwhelming MDR to ampicillin, cefotaxime, cefepime, chloramphenicol, and nalidixic acid [46]. Due to the difference between the detection rates of ESBL-SE and non–ESBL-SE isolates resistant to some first-line antibiotics (e.g., cefotaxime, ceftazidime, ampicillin, chloramphenicol, and nalidixic acid), it remains challenging to treat human infections by ESBL-producing bacteria in the clinic. The presence of MDR phenotypes among ESBL-SE isolates may be due to inappropriate combination of multiple classes of broad-spectrum antibiotics [22]. Consequently, ESBL-producing *Salmonella* could be considered an indicator for the observation of MDR *Salmonella* in the clinical treatment of salmonellosis.

Among the 233 ESBL-SE isolates in the present study, the most commonly detected ESBL-encoding genes were *bla*_CTX-Ms_, followed by *bla*_TEMs_ (Table 2). The high prevalence of *bla*_CTX-M_ genes among ESBL-producing *Salmonella* is similar to the previous findings from *Salmonella* and *E. coli* in patients, animals, and foods in China [19,20,22]. Here, *bla*_CTX-M-55_ was the most prevalent *bla*_CTX-M_ subtype detected, and its detection rate in the patients from Shanghai increased remarkably since 2011 (Table 2). In 2011, *bla*_CTX-M-55_ was first reported from human *Salmonella* isolates in China [47]. Subsequently, it was found that *bla*_CTX-M-55_ contributes to the spread of antibiotic resistance among bacteria in foods, animals, and humans [15,48,49,50,51]. Although other *bla*_CTX-M_ variants, such as *bla*_CTX-M-3_, *bla*_CTX-M-15_, *bla*_CTX-M-64_, *bla*_CTX-M-79_, and *bla*_CTX-M-123_ were rarely detected in our study, their presence in human patients suggests the occurrence of the diversity and evolution of *bla*_CTX-M_ in clinical treatment.

When compared with *bla*_CTX-M-55_-negative isolates, *bla*_CTX-M-55_-postitive isolates were more frequently to first-, third-, and fourth-generation cephalosporins (Table 3). This suggests that *bla*_CTX-M-55_-carrying ESBL-SE isolates have a strong activity to hydrolyze cephalosporins. If other antibiotic resistance genes are co-transferred with *bla*_CTX-M-55_, it would render clinical salmonellosis treatment even more difficult. A previous study also reported that *bla*_CTX-M_-postitive *S.* Enteritidis strains recovered from patients and foods in Shanghai were resistant to quinolones due to *gyr*A gene mutation [19]. Cefepime is the first fourth-generation cephalosporin permitted to be used in China for treating serious gram-negative infections [52]. Based on our results that ESBL-SE isolates were susceptible to cefoxitin, we recommend that clinicians in Shanghai use cefoxitin for empirical treatment of ESBL-SE infections, while cefepime should be used with caution to prolong the effectiveness of new antibiotics (Table S4). In the present study, PFGE revealed that most *bla*_CTX-M-55_-postitive ESBL-SE isolates had similar DNA profiles. Additionally, even though *bla*_CTX-M-55_-carrying ESBL-SE isolates were recovered from different years, districts, hospitals, and outpatients, the PFGE patterns of some isolates in types B1-1, B3-1, and B1-2 exhibited 100% genomic similarity (Figure 5, Table S5). It is, therefore, most possible that the presence of the *bla*_CTX-M-55_ gene in these isolates was attributed to clonal transfer of *bla*_CTX-M-55_-postitive ESBL-SE within hospitals or contaminated foods. The transmission of the *bla*_CTX-M-55_ gene to *Enterobacteriaceae* through ESBL-producing *Salmonella* in vitro would be one of the main hazards for the dissemination of antibiotic resistance; some *Salmonella* isolates have already been described as having acquired *bla*_CTX-M-55_-conferred resistance [49,53]. In particular, the PFGE pattern of one bloodborne ESBL-SE isolate (isolate 114) in pattern B4-1 was identical to that of three isolates recovered from fecal samples (isolate 273, 244, 225; Table S5). This result highlights the importance of paying more attention to nosocomial infection. Further, the results of the conjugation experiment (Table S6) confirmed previous reports that the *bla*_CTX-M-55_ gene in ESBL-SE strains can be easily transferred into *Enterobacteriaceae* recipient strains, irrespective of whether they are wild-type or engineered strains [49]. Our results indicate that the *bla*_CTX-M-55_ gene has the potential to disseminate to other bacterial reservoirs.

In conclusion, we confirm that 233 (79.8%) out of 292 *S.* Enteritidis isolates recovered from human patients in Shanghai (2006–2014) were ESBL-positive. From the 233 ESBL-SE isolates, eight ESBL genotypes were detected: *bla*_CTX-M-55_, *bla*_TEM-1_, *bla*_CTX-M-64_, *bla*_CTX-M-123_, *bla*_TEM-214_, *bla*_CTX-M-3_, *bla*_CTX-M-79_, and *bla*_CTX-M-15_. Notably, our findings highlight the presence of *bla*_CTX-M-55_ as the most common genotype in *S.* Enteritidis among human patients in Shanghai, China. The high genomic similarities of *bla*_CTX-M-55_-postitive ESBL-SE isolates of clinical origin pose a vital threat to public health, as the gene can be eventually transferred to the entire food chain. Cephalosporins, especially cefotaxime, should be used with caution for empirical treatment of Salmonella infections.

## Figures and Tables

**Table 1:** Clinical background information of ESBL-producing *S*. Enteritidis (ESBL-SE) and non-ESBL-producing *S*. Enteritidis (non-ESBL-SE) isolates ^1^.

Characteristic	No. (%) of ESBL-SE Isolates (*n* = 233)	No. (%) of Non-ESBL-SE Isolates (*n* = 59)	Unadjusted OR (95% CI) ^1^
Year			
2006–2010	6 (2.6)	22 (37.3)	1.0 (ref)
2011	19 (8.2)	21 (35.6)	3.3 (1.1–9.9)
2012	46 (19.7)	2 (3.4)	84.3 (15.7–452.1)
2013	131 (56.2)	8 (13.6)	60.0 (19.0–189.8)
2014	31 (13.3)	6 (10.2)	18.9 (5.4–66.6)
Source			
General outpatient	33 (14.2)	4 (6.8)	1.0 (ref)
Intestinal outpatient	132 (56.7)	47 (79.7)	0.3 (0.1–1.0)
Other outpatient	45 (19.3)	8 (13.6)	0.7 (0.2–2.5)
Others	23 (9.9)	0 (0.0)	
District			
Minhang district	109 (46.8)	22 (37.3)	1.0 (ref)
Jing’an district	53 (22.7)	5 (8.5)	2.1 (0.8–6.0)
Jinshan district	14 (6.0)	7 (11.9)	0.4 (0.2–1.1)
Huangpu district	8 (3.4)	10 (16.9)	0.2 (0.06–0.5)
Changning district	9 (3.9)	8 (13.6)	0.2 (0.08–0.7)
Others	40 (17.2)	7 (11.9)	1.2 (0.5–2.9)
Hospital			
Public hospital	79 (33.9)	33 (55.9)	1.0 (ref)
Pediatric hospital	126 (54.1)	25 (42.4)	2.1 (1.2–3.9)
Community hospital	14 (6.0)	1 (1.7)	5.9 (0.8–46.9)
Others	14 (6.0)	0 (0.0)	

^1^ OR, odds ratio; 95% CI, confidence interval; ref, reference group. All isolates tested include ESBL-SE and non-ESBL-SE.

**Table 2:** The prevalence of ESBL-encoding genes of ESBL-producing *S.* Enteritidis isolates recovered from different years.

ESBL-Encoding Gene	No. (%) of Isolates by Year					Total No. (%) (*n* = 233)
	2006–2010 (*n* = 6)	2011 (*n* = 19)	2012 (*n* = 46)	2013 (*n* = 131)	2014 (*n* = 31)	
***bla*_CTX-M_**						
*bla*_CTX-M__-3_	0 (0.0)	1 (5.3)	2 (4.3)	0 (0.0)	0 (0.0)	3 (1.3)
*bla*_CTX-M__-15_	0 (0.0)	0 (0.0)	1 (2.2)	0 (0.0)	0 (0.0)	1 (0.4)
*bla*_CTX-M__-55_	0 (0.0)	13 (68.4)	28 (60.9)	90 (68.7)	21 (67.7)	152 (65.2)
*bla*_CTX-M__-64_	0 (0.0)	0 (0.0)	4 (8.7)	0 (0.0)	0 (0.0)	4 (1.7)
*bla*_CTX-M__-79_	0 (0.0)	0 (0.0)	1 (2.2)	1 (0.8)	0 (0.0)	2 (0.9)
*bla*_CTX-M__-123_	1 (16.7)	1 (5.3)	1 (2.2)	0 (0.0)	1 (3.2)	4 (1.7)
Total	1 (16.7)	15 (78.9)	37 (80.4)	91 (69.5)	22 (71.0)	166 (71.2)
***bla*_TEM_**						
*bla*_TEM-1_	1 (16.7)	12 (63.2)	26 (56.5)	14 (10.7)	3 (9.7)	56 (24.0)
*bla*_TEM-214_	0 (0.0)	3 (15.8)	0 (0.0)	0 (0.0)	0 (0.0)	3 (1.3)
Total	1 (16.7)	15 (78.9)	26 (56.5)	14 (10.7)	3 (9.7)	59 (25.3)

**Table 3:** The minimum inhibitory concentrations (MICs) of cephalosporins for clinical ESBL-producing *S.* Enteritidis (ESBL-SE) isolates (*n* = 233).

Cephalosporin	*bla*_CTX-M-55_-Positive ESBL-SE (*n* = 152)			*bla*_CTX-M-55_-Negative ESBL-SE (*n* = 81)			*p* Value ^1^
	MIC50 (μg/mL)	MIC90 (μg/mL)	Resistance (%)	MIC50 (μg/mL)	MIC90 (μg/mL)	Resistance (%)	
Cefotaxime	64	64	100.0	64	64	100.0	-
Ceftazidime	16	32	91.5	16	32	67.9	0.0 **
Ceftriaxone	128	128	98.0	128	128	92.6	0.04 *
Cefepime	16	16	75.0	16	16	58.0	0.008 **
Cefazolin	16	16	98.0	16	16	92.6	0.04 *
Cefpodoxime	32	32	98.0	32	32	92.6	0.04 *
Cefoxitin	4	4	0.7	4	8	2.5	0.2

^1^ *, *p* < 0.05; **, *p* < 0.01 (by χ^2^ test).

## Data Availability

The data presented in this study are available in Supplementary Materials.

## Supplementary Materials

The following are available online at https://www.mdpi.com/2076-2607/9/2/260/s1, Figure S1: The number and detection rate of ESBL-producing *S.* Enteritidis (ESBL-SE) isolates recovered from different years (*n* = 292). Table S1: Primers used for the detection of β-lactamase genes among Salmonella enterica serovar Enteritidis isolates in this study. Table S2: The donor and recipient strains of ESBL-encoding gene used for conjugation experiment. Black squares denote the presence of resistance to a given antimicrobial agent. Table S3: Antibiotic resistance of *S.* Enteritidis isolates recovered from human patients in Shanghai, 2006–2014 (*n* = 292). Table S4: Distribution of the minimum inhibitory concentrations (MICs) of seven cephalosporins against ESBL-producing *S.* Enteritidis isolates (*n* = 233). Table S5: PFGE pattern, clinical background information, and antibiotic resistance of selected ESBL-producing *S*. Enteritidis isolates (*n* = 113). Table S6: Antibiotic resistance profiles of donor strains, recipient strains, and transconjugants, and the conjugation frequency of ESBL-encoding genes. Black squares denote the presence of resistance to a given antimicrobial agent.
