# Supplementary material for: Epidemiology and Characterization of CTX-M-55-Type Extended-Spectrum β-Lactamase-Producing Salmonella enterica Serovar Enteritidis Isolated from Patients in Shanghai, China
Source: Microorganisms. 2021 Jan 27;9(2):260. doi: 10.3390/microorganisms9020260 (PMC7912593; doi:10.3390/microorganisms9020260)
Supplement: Supplementary file 1 [file microorganisms-09-00260-s001.pdf]

Supplementary Materials for

Epidemiology and characterization of CTX-M-55-type extended-spectrum β-lactamase–producing *Salmonella enterica* serovar Enteritidis isolated from patients in Shanghai, China

Chenyang Cao[1], Qinya Niu[1], Jia Chen[2], Xuebin Xu[3], Huanjing Sheng[1], Shenghui Cui[4], Bin Liu[1] and Baowei Yang[1,*]

[1] College of Food Science and Engineering, Northwest A&F University, Yangling 712100, China
[2] College of Chemical Technology, Shijiazhuang University, Shijiazhuang 050035, China
[3] Shanghai Municipal Center for Disease Control & Prevention, Shanghai 200336, China
[4] National Institutes for Food and Drug Control, Beijing 100050, China

*Correspondence: ybwsheng@nwafu.edu.cn; Tel.: +86-29-87092486; Fax: +86-29-87092486

Table of Content

Figure S1 The number and detection rate of ESBL-producing *S.* Enteritidis (ESBL-SE) isolates recovered from different years (n = 292).

Table S1 Primers used for the detection of β-lactamase genes among *Salmonella enterica* serovar Enteritidis isolates in this study.

Table S2 The donor and recipient strains of ESBL-encoding gene used for conjugation experiment. Black squares denote the presence of resistance to a given antimicrobial agent.

Table S3 Antibiotic resistance of *S.* Enteritidis isolates recovered from human patients in Shanghai, 2006–2014 (n = 292).

Table S4 Distribution of the minimum inhibitory concentrations (MICs) of seven cephalosporins against ESBL-producing *S.* Enteritidis isolates (n = 233).

Table S5 PFGE pattern, clinical background information, and antibiotic resistance of selected ESBL-producing *S.* Enteritidis isolates (n = 113).

Table S6 Antibiotic resistance profiles of donor strains, recipient strains, and transconjugants, and the conjugation frequency of ESBL-encoding genes. Black squares denote the presence of resistance to a given antimicrobial agent.

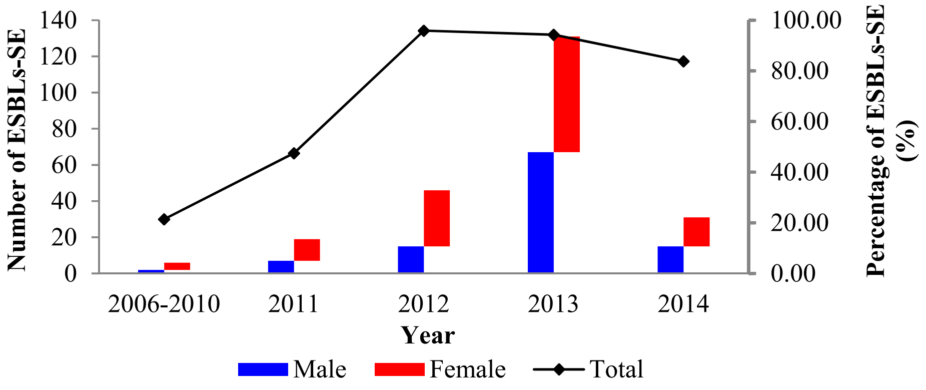

Figure S1. The number and detection rate of ESBL-producing *S.* Enteritidis (ESBL-SE) isolates recovered from different years (n = 292).

Table S1 Primers used for the detection of β-lactamase genes among *Salmonella enterica* serovar Enteritidis isolates in this study.

Gene	Primer	Sequence (5'–3')	Product (bp)	Reference
bla$_{CTX-M}$	*bla*$_{CTX-M}$-F	GAGTTTCCCCATTCCGTTTC	880	[1]
	bla$_{CTX-M}$-R	CAGAATAAGGAATCCCATGGTT		
bla$_{TEM}$	*bla*$_{TEM}$-F	ATGAGTATTCAACATTTCCG	964	[2]
	bla$_{TEM}$-R	ACCAATGCTTAATCAGTGAG		
bla$_{CMY}$	*bla*$_{CMY}$-F	GACAGCCTCTTTCTCCACA	1000	[3]
	bla$_{CMY}$-R	TGGAACGAAGGCTACGTA		
bla$_{ACC}$	*bla*$_{ACC}$-F	AGCCTCAGCAGCCGGTTAC	818	[2]
	bla$_{ACC}$-R	GAAGCCGTTAGTTGATCCGG		
bla$_{SHV}$	*bla*$_{SHV}$-F	TTCGCCTGTGTATTATCTCCCTG	854	[2]
	bla$_{SHV}$-R	TTAGCGTTGCCAGTGCTCG		
bla$_{VEB}$	*bla*$_{VEB}$-F	GATAGGAGTACAGACATATG	914	[1]
	bla$_{VEB}$-R	TTTATTCAAATAGTAATTCCACG		
bla$_{PER}$	*bla*$_{PER}$-F	ATGAATGTCATCACAAAATG	927	[1]
	bla$_{PER}$-R	TCAATCCGGACTCACT		
bla$_{GES}$	*bla*$_{GES}$-F	ATGCGCTTCATTCACGCAC	864	[1]
	bla$_{GES}$-R	CTATTTGTCCGTGCTCAGG		
bla$_{PSE}$	*bla*$_{PSE}$-F	AATGGCAATCAGCGCTTCCC	598	[3]
	bla$_{PSE}$-R	GGGGCTTGATGCTCACTACA		
bla$_{OXA}$	*bla*$_{OXA}$-F	ACCAGATTCAACTTTCAA	590	[4]
	bla$_{OXA}$-R	TCTTGGCTTTTATGCTTG		

Table S2 The donor and recipient strains of ESBL-encoding gene used for conjugation experiment. Black squares denote the presence of resistance to a given antimicrobial agent.

	**Strain**	**Organism**	**Source**	**Sample**	**PFGE Pattern**	**Year**	**ESBL-encoding gene**	**AMP**	**AMC**	**CTX**	**CAZ**	**GEN**	**TET**	**CIP**	**NAL**	**SXT**	**CHL**
Donor	71	*Salmonella* Enteritidis	Clinical	Feces	A4	2011	*bla*$_{CTX\text{-}M\text{-}55}$/*bla*$_{TEM\text{-}1}$	■		■	■		■		■	■	
	86	*Salmonella* Enteritidis	Clinical	Feces	A35	2012	*bla*$_{CTX\text{-}M\text{-}55}$	■		■	■		■		■	■	■
	122	*Salmonella* Enteritidis	Clinical	Feces	A19	2013	*bla*$_{CTX\text{-}M\text{-}55}$	■		■	■				■	■	
Recipient	20	*Salmonella* Enteritidis	Clinical	Feces		2009	-	■	■	■		■	■		■	■	■
	1-22	*Escherichia coli*	Food	Chicken		2016	-	■					■	■	■	■	■
	C600	*Escherichia coli*					-								■		

AMP: Ampicillin; AMC: Amoxicillin-Clavulanic acid; CTX: Cefotaxime; CAZ: Ceftazidime; GEN : Gentamicin; TET: Tetracycline; CIP: Ciprofloxacin; NAL : Nalidixic acid; SXT: Sulfamethoxazole/Trimethoprim; CHL: Chloramphenicol.

Table S3 Antibiotic resistance of *S.* Enteritidis isolates recovered from human patients in Shanghai, 2006–2014 (n = 292).

Antibiotic	Total No. (%) (n = 292)	Resistance to individual antibiotics by year (%)[a]				
		2006–2010 (n = 28)	2011 (n = 40)	2012 (n = 48)	2013 (n = 139)	2014 (n = 37)
Cefotaxime	284 (97.3)[a]	28 (100.0)[a]	32 (80.0)[b]	48 (100.0)[a]	139 (100.0)[a]	37 (100.0)[a]
Ceftazidime	251 (86.0)[b]	9 (32.1)[c]	5 (12.5)[c]	41 (85.4)[b]	130 (93.5)[b]	34 (91.9)[a]
Ampicillin	250 (85.6)[b]	24 (85.7)[b]	5 (12.5)[c]	47 (97.9)[a]	138 (99.3)[a]	37 (100.0)[a]
Nalidixic acid	219 (75.0)[c]	28 (100.0)[a]	0 (0.0)[d]	47 (97.9)[a]	138 (99.3)[a]	37 (100.0)[a]
Sulfisoxazole	140 (48.0)[e]	21 (75.0)[b]	2 (5.0)[cd]	28 (58.3)[c]	69 (49.6)[c]	20 (54.1)[b]
Streptomycin	168 (57.5)[d]	19 (67.9)[b]	36 (90.0)[ab]	28 (58.3)[c]	68 (48.9)[c]	17 (46.0)[bc]
Tetracycline	130 (44.5)[e]	7 (25.0)[c]	29 (72.5)[b]	20 (41.7)[c]	65 (46.8)[c]	9 (24.3)[c]
Chloramphenicol	79 (27.1)[f]	3 (10.7)[c]	0 (0.0)[d]	8 (16.7)[d]	60 (43.2)[c]	8 (21.6)[c]
Sulfamethoxazole/ Trimethoprim	75 (25.7)[f]	7 (25.0)[c]	38 (95.0)[a]	4 (8.3)[d]	17 (12.2)[d]	9 (24.3)[c]
Amoxicillin/Clavu lanate	21 (7.2)[h]	5 (17.9)[c]	0 (0.0)[d]	5 (10.4)[d]	6 (4.3)[e]	5 (13.5)[cd]
Ciprofloxacin	33 (11.3)[h]	21 (75.0)[b]	5 (12.5)[c]	0 (0.0)[e]	6 (4.3)[e]	1 (2.7)[d]
Ofloxacin	11 (3.8)[i]	3 (10.7)[c]	4 (10.0)[c]	0 (0.0)[e]	4 (2.9)[e]	0 (0.0)[d]
Trimethoprim	51 (17.5)[g]	7 (25.0)[c]	29 (72.5)[b]	3 (6.3)[de]	4 (2.9)[e]	8 (21.6)[c]
Gentamicin	53 (18.6)[g]	10 (35.7)[c]	27 (67.5)[b]	6 (12.5)[d]	2 (1.4)[e]	8 (21.6)[c]

[a] In each column, the resistance rates of individual antibiotics sharing the same lowercase letters show no significant difference ($P > 0.05$).

Table S4 Distribution of the minimum inhibitory concentrations (MICs) of seven cephalosporins against ESBL-producing *S.* Enteritidis isolates (n = 233).

Cephalosporins	Breakpoint (μg/mL)	Distribution of MICs (μg/mL)					Resistance (%)
		128 (%)	64 (%)	32 (%)	16 (%)	4 (%)	
Cefotaxime	≥ 4		202 (86.7)	17 (7.3)	5 (2.2)	9 (3.9)	233 (100.0)
Ceftazidime	≥ 16	8 (3.4)	7 (3.0)	67 (28.8)	112 (48.1)		194 (83.3)
Ceftriaxone	≥ 4	191 (82.0)	25 (10.7)	8 (3.4)			224 (96.1)
Cefepime	≥ 16				161 (69.1)		161 (69.1)
Cefazolin	≥ 8				224 (96.1)		224 (96.1)
Cefpodoxime	≥ 8			224 (96.1)			224 (96.1)
Cefoxitin	≥ 32		2 (0.9)	1 (0.4)			3 (1.3)

Table S5 PFGE pattern, clinical background information, and antibiotic resistance of selected ESBL-producing *S.* Enteritidis isolates (*n* = 113).

PFGE pattern	Strain	Year	District[a]	Hospital	Hospital type	Source	Sample	Age[b]	ESBL-encoding gene
B1-1	137	2013	C	C2	Public hospital	Intestinal outpatient	Feces	58 Y	$bla_{CTX-M-55}$/bla_{TEM-1}
B1-1	138	2013	C	C5	Community hospital	Intestinal outpatient	Feces	10 Y	$bla_{CTX-M-55}$
B1-1	139	2013	C	C2	Public hospital	Intestinal outpatient	Feces	9 Y	$bla_{CTX-M-55}$
B1-1	140	2013	C	C5	Community hospital	Intestinal outpatient	Feces	79 Y	$bla_{CTX-M-55}$
B1-1	141	2013	C	C6	Community hospital	Intestinal outpatient	Feces	44 Y	$bla_{CTX-M-55}$
B1-1	277	2014	F	F3	Community hospital	Intestinal outpatient	Feces	62 Y	$bla_{CTX-M-55}$
B1-1	278	2014	F	F5	Public hospital	Intestinal outpatient	Feces	24 Y	$bla_{CTX-M-55}$
B1-1	265	2014	D	D3	Public hospital	General outpatient	Feces	25 Y	$bla_{CTX-M-55}$
B1-1	268	2014	A	A5	Public hospital	Intestinal outpatient	Feces	43 Y	$bla_{CTX-M-55}$
B1-1	270	2014	I	I1	Public hospital	Intestinal outpatient	Feces	47 Y	$bla_{CTX-M-55}$
B1-1	271	2014	H	H2	Public hospital	Intestinal outpatient	Feces	66 Y	$bla_{CTX-M-55}$
B1-1	250	2013	F	F5	Public hospital	Intestinal outpatient	Feces	21 Y	$bla_{CTX-M-55}$
B1-1	253	2013	F	F4	Public hospital	Intestinal outpatient	Feces	31 Y	$bla_{CTX-M-55}$
B1-1	240	2013	A	A2	Pediatric Hospital	Other outpatient	Feces	7 M	$bla_{CTX-M-55}$
B1-1	241	2013	A	A2	Pediatric Hospital	Other outpatient	Feces	8 M	$bla_{CTX-M-55}$
B1-1	246	2013	A	A2	Pediatric Hospital	Other outpatient	Feces	6 Y	$bla_{CTX-M-55}$
B1-1	196	2013	A	A2	Pediatric Hospital	General outpatient	Feces	8 M	$bla_{CTX-M-55}$
B1-1	193	2013	A	A2	Pediatric Hospital	General outpatient	Feces	4 Y	$bla_{CTX-M-55}$

PFGE pattern	Strain	Year	District[a]	Hospital	Hospital type	Source	Sample	Age[b]	ESBL-encoding gene
B1-1	192	2013	A	A2	Pediatric Hospital	Other outpatient	Feces	3 Y	$bla_{\text{CTX-M-55}}$
B1-1	190	2013	A	A2	Pediatric Hospital	Other outpatient	Feces	1 Y	$bla_{\text{CTX-M-55}}$
B1-1	188	2013	A	A2	Pediatric Hospital	Other outpatient	Feces	2 Y	$bla_{\text{CTX-M-55}}$
B1-1	185	2013	A	A2	Pediatric Hospital	Other outpatient	Feces	1 Y	$bla_{\text{CTX-M-55}}$
B1-1	89	2012	G	G3	Community hospital	Intestinal outpatient	Feces	60 Y	$bla_{\text{CTX-M-55}}/bla_{\text{TEM-1}}$
B1-1	97	2012	K	K1	Public hospital	Other	Feces	60 Y	$bla_{\text{CTX-M-55}}/bla_{\text{TEM-1}}$
B1-1	233	2013	A	A2	Pediatric Hospital	General outpatient	Feces	8 M	$bla_{\text{CTX-M-55}}$
B1-1	234	2013	A	A2	Pediatric Hospital	Other outpatient	Feces	3 Y	$bla_{\text{CTX-M-55}}$
B1-1	237	2013	A	A2	Pediatric Hospital	Other outpatient	Feces	4 Y	$bla_{\text{CTX-M-55}}$
B1-1	223	2013	B	B1	Pediatric Hospital	Intestinal outpatient	Feces	3 Y	$bla_{\text{CTX-M-55}}$
B1-1	216	2013	B	B2	Public hospital	Intestinal outpatient	Feces	56 Y	$bla_{\text{CTX-M-55}}$
B1-1	217	2013	B	B2	Public hospital	Intestinal outpatient	Feces	26 Y	$bla_{\text{CTX-M-55}}$
B1-1	218	2013	B	B2	Public hospital	Intestinal outpatient	Feces	56 Y	$bla_{\text{CTX-M-55}}$
B1-1	206	2013	B	B1	Pediatric Hospital	Intestinal outpatient	Feces	3 Y	$bla_{\text{CTX-M-55}}$
B1-1	208	2013	B	B1	Pediatric Hospital	Intestinal outpatient	Feces	8 M	$bla_{\text{CTX-M-55}}$
B1-1	209	2013	B	B1	Pediatric Hospital	Intestinal outpatient	Feces	3 Y	$bla_{\text{CTX-M-55}}$
B1-1	200	2013	A	A2	Pediatric Hospital	Other	Feces	5 M	$bla_{\text{CTX-M-55}}$
B1-1	94	2012	E	E3	Other	Other	Feces	8 Y	$bla_{\text{CTX-M-55}}$
B1-1	95	2012	E	E1	Public hospital	Intestinal outpatient	Feces	14 Y	$bla_{\text{CTX-M-55}}$
B1-1	106	2012	A	A2	Pediatric Hospital	General outpatient	Feces	2 Y	$bla_{\text{CTX-M-55}}$
B1-1	183	2013	A	A2	Pediatric Hospital	Other outpatient	Feces	1 Y	$bla_{\text{CTX-M-55}}$

PFGE pattern	Strain	Year	District[a]	Hospital	Hospital type	Source	Sample	Age[b]	ESBL-encoding gene
B1-1	207	2013	B	B1	Pediatric Hospital	Intestinal outpatient	Feces	2 Y	$bla_{CTX-M-55}$
B1-1	212	2013	B	B2	Public hospital	Intestinal outpatient	Feces	25 Y	$bla_{CTX-M-55}$
B1-1	275	2014	D	D1	Public hospital	Intestinal outpatient	Feces	26 Y	$bla_{CTX-M-55}$
B1-2	274	2014	A	A2	Pediatric Hospital	Other	Feces	1 Y	$bla_{CTX-M-55}$
B1-2	199	2013	A	A2	Pediatric Hospital	General outpatient	Feces	4 Y	$bla_{CTX-M-55}$
B1-2	198	2013	A	A2	Pediatric Hospital	General outpatient	Feces	2 Y	$bla_{CTX-M-55}$
B1-2	135	2013	C	C4	Public hospital	Intestinal outpatient	Feces	1 Y	$bla_{CTX-M-55}$
B1-2	107	2012	A	A2	Pediatric Hospital	General outpatient	Feces	5 Y	$bla_{CTX-M-55}$
B1-2	220	2013	B	B1	Pediatric Hospital	Intestinal outpatient	Feces	3 Y	$bla_{CTX-M-55}$
B1-2	221	2013	B	B1	Pediatric Hospital	Intestinal outpatient	Feces	17 Y	$bla_{CTX-M-55}$
B1-2	222	2013	B	B1	Pediatric Hospital	Intestinal outpatient	Feces	17 Y	$bla_{CTX-M-55}$
B1-2	214	2013	B	B1	Pediatric Hospital	Intestinal outpatient	Feces	1 Y	$bla_{CTX-M-55}/bla_{TEM-1}$
B1-2	215	2013	B	B2	Public hospital	Intestinal outpatient	Feces	50 Y	$bla_{CTX-M-55}$
B1-5	166	2013	G	G1	Public hospital	Intestinal outpatient	Feces	55 Y	$bla_{CTX-M-55}$
B1-5	108	2012	A	A2	Pediatric Hospital	General outpatient	Feces	3 Y	$bla_{CTX-M-55}$
B1-5	279	2014	J	J2	Public hospital	Intestinal outpatient	Feces	34 Y	$bla_{CTX-M-55}$
B1-12	142	2013	A	A5	Public hospital	Intestinal outpatient	Feces	46 Y	$bla_{CTX-M-55}$
B1-12	296	2014	G	G3	Community hospital	Intestinal outpatient	Feces	26 Y	$bla_{CTX-M-55}$

PFGE pattern	Strain	Year	District[a]	Hospital	Hospital type	Source	Sample	Age[b]	ESBL-encoding gene
B2-9	295	2014	G	G1	Public hospital	Intestinal outpatient	Feces	64 Y	*bla*$_{CTX-M-55}$
B2-9	302	2014	B	B1	Pediatric Hospital	Intestinal outpatient	Feces	11 M	*bla*$_{CTX-M-55}$
B2-9	290	2013	A	A2	Pediatric Hospital	Intestinal outpatient	Feces	4 Y	*bla*$_{CTX-M-55}$/*bla*$_{TEM-1}$
B2-10	38	2011	J	J1	Public hospital	Intestinal outpatient	Feces	27 Y	*bla*$_{CTX-M-55}$/*bla*$_{TEM-1}$
B2-10	39	2011	G	G1	Public hospital	Intestinal outpatient	Feces	23 Y	*bla*$_{CTX-M-55}$/*bla*$_{TEM-1}$
B2-10	41	2011	A	A5	Pediatric Hospital	Intestinal outpatient	Feces	4 Y	*bla*$_{CTX-M-55}$/*bla*$_{TEM-1}$
B2-10	50	2011	C	C1	Public hospital	Intestinal outpatient	Feces	2 Y	*bla*$_{CTX-M-55}$/*bla*$_{TEM-214}$
B2-10	65	2011	G	G3	Public hospital	Intestinal outpatient	Feces	59 Y	*bla*$_{CTX-M-55}$/*bla*$_{TEM-1}$
B2-10	66	2012	B	B3	Public hospital	Intestinal outpatient	Feces	58 Y	*bla*$_{CTX-M-55}$
B2-10	76	2012	A	A2	Pediatric Hospital	Intestinal outpatient	Feces	4 Y	*bla*$_{CTX-M-55}$
B2-10	79	2012	A	A2	Pediatric Hospital	Intestinal outpatient	Feces	2 Y	*bla*$_{CTX-M-55}$/*bla*$_{TEM-1}$
B2-10	280	2014	J	J3	Public hospital	Intestinal outpatient	Feces	34 Y	*bla*$_{CTX-M-55}$
B2-11	87	2012	B	B1	Pediatric Hospital	Intestinal outpatient	Feces	17 D	*bla*$_{CTX-M-55}$/*bla*$_{TEM-1}$
B2-11	88	2012	B	B1	Pediatric Hospital	Intestinal outpatient	Feces	2 Y	*bla*$_{CTX-M-55}$/*bla*$_{TEM-1}$
B2-11	91	2012	F	F3	Community hospital	Intestinal outpatient	Feces	54 Y	*bla*$_{CTX-M-55}$/*bla*$_{TEM-1}$
B2-11	93	2012	H	H2	Public hospital	Intestinal outpatient	Feces	49 Y	*bla*$_{CTX-M-55}$/*bla*$_{TEM-1}$
B2-11	168	2013	D	D1	Public hospital	Intestinal outpatient	Feces	64 Y	*bla*$_{CTX-M-55}$
B3-1	184	2013	A	A2	Pediatric Hospital	Other outpatient	Feces	1 Y	*bla*$_{CTX-M-55}$

PFGE pattern	Strain	Year	District[a]	Hospital	Hospital type	Source	Sample	Age[b]	ESBL-encoding gene
B3-1	124	2013	C	C3	Public hospital	Intestinal outpatient	Feces	55 Y	*bla*$_{CTX-M-55}$
B3-1	125	2013	C	C4	Public hospital	Intestinal outpatient	Feces	17 Y	*bla*$_{CTX-M-55}$
B3-1	126	2013	A	-	Other	Other	Feces	45 Y	*bla*$_{CTX-M-55}$
B3-1	151	2013	B	-	Other	Other	Feces	33 Y	*bla*$_{CTX-M-55}$
B3-1	148	2013	B	-	Other	Other	Feces	21 Y	*bla*$_{CTX-M-55}$
B3-1	150	2013	B	-	Other	Other	Feces	24 Y	*bla*$_{CTX-M-55}$
B3-1	152	2013	B	-	Other	Other	Feces	23 Y	*bla*$_{CTX-M-55}$
B3-1	153	2013	B	B2	Public hospital	Intestinal outpatient	Feces	30 Y	*bla*$_{CTX-M-55}$
B3-1	154	2013	B	B2	Public hospital	Intestinal outpatient	Feces	27 Y	*bla*$_{CTX-M-55}$/*bla*$_{TEM-1}$
B3-1	155	2013	B	B2	Public hospital	Intestinal outpatient	Feces	30 Y	*bla*$_{CTX-M-55}$
B3-1	156	2013	A	A2	Pediatric Hospital	Other outpatient	Feces	2 Y	*bla*$_{CTX-M-55}$
B3-1	159	2013	A	A2	Pediatric Hospital	Other outpatient	Feces	1 Y	*bla*$_{CTX-M-55}$
B3-1	161	2013	A	A2	Pediatric Hospital	Other outpatient	Feces	1 Y	*bla*$_{CTX-M-55}$
B3-1	162	2013	A	A2	Pediatric Hospital	Other outpatient	Feces	11 M	*bla*$_{CTX-M-55}$
B3-1	163	2013	A	A2	Pediatric Hospital	General outpatient	Feces	2 Y	*bla*$_{CTX-M-55}$
B3-1	144	2013	B	-	Other	Other	Feces	32 Y	*bla*$_{CTX-M-55}$
B3-1	145	2013	B	-	Other	Other	Feces	22 Y	*bla*$_{CTX-M-55}$/*bla*$_{TEM-1}$
B3-1	146	2013	B	-	Other	Other	Feces	27 Y	*bla*$_{CTX-M-55}$
B3-1	147	2013	B	-	Other	Other	Feces	34 Y	*bla*$_{CTX-M-55}$
B3-1	167	2013	D	D2	Public hospital	Intestinal outpatient	Feces	81 Y	*bla*$_{CTX-M-55}$
B3-1	169	2013	K	K1	Public hospital	Other outpatient	Feces	22 Y	*bla*$_{CTX-M-55}$

PFGE pattern	Strain	Year	District[a]	Hospital	Hospital type	Source	Sample	Age[b]	ESBL-encoding gene
B3-1	170	2013	L	L1	Public hospital	Intestinal outpatient	Feces	84 Y	*bla*$_{CTX-M-55}$
B3-1	172	2013	E	E1	Public hospital	Other outpatient	Feces	72 Y	*bla*$_{CTX-M-55}$/*bla*$_{TEM-1}$
B3-1	173	2013	E	E1	Public hospital	Intestinal outpatient	Feces	76 Y	*bla*$_{CTX-M-55}$/*bla*$_{TEM-1}$
B3-1	293	2014	J	J2	Public hospital	Intestinal outpatient	Feces	65 Y	*bla*$_{CTX-M-55}$
B3-1	294	2014	G	G1	Public hospital	Intestinal outpatient	Feces	62 Y	*bla*$_{CTX-M-55}$
B3-1	281	2014	F	F3	Community hospital	Intestinal outpatient	Feces	72 Y	*bla*$_{CTX-M-55}$
B3-1	291	2013	A	A2	Pediatric Hospital	Intestinal outpatient	Feces	1 Y	*bla*$_{CTX-M-55}$
B3-1	195	2013	A	A2	Pediatric Hospital	General outpatient	Feces	8 M	*bla*$_{CTX-M-55}$
B3-2	84	2012	A	A2	Pediatric Hospital	Intestinal outpatient	Feces	3 Y	*bla*$_{CTX-M-55}$/*bla*$_{TEM-1}$
B3-2	69	2011	A	A2	Pediatric Hospital	Other	Feces	4 Y	*bla*$_{CTX-M-55}$/*bla*$_{TEM-1}$
B3-2	122	2013	A	A1	Public hospital	Intestinal outpatient	Feces	46 Y	*bla*$_{CTX-M-55}$
B4-1	273	2014	A	A2	Pediatric Hospital	Other outpatient	Feces	7 M	*bla*$_{CTX-M-55}$/*bla*$_{TEM-1}$
B4-1	244	2013	A	A2	Pediatric Hospital	Other outpatient	Feces	1 Y	*bla*$_{CTX-M-55}$/*bla*$_{TEM-1}$
B4-1	225	2013	B	B1	Pediatric Hospital	Intestinal outpatient	Feces	2 Y	*bla*$_{CTX-M-55}$
B4-1	114	2012	B	B3	Public hospital	Other	Blood	19 Y	*bla*$_{CTX-M-55}$
B5-2	100	2012	A	A2	Pediatric Hospital	Intestinal outpatient	Feces	4 M	*bla*$_{CTX-M-55}$/*bla*$_{TEM-1}$
B5-2	104	2012	A	A2	Pediatric Hospital	Intestinal outpatient	Feces	1 Y	*bla*$_{CTX-M-55}$/*bla*$_{TEM-1}$

[a] The district code is shown in Figure 1.

[b] Y: Year; M: Month; D: Day.

Table S6 Antibiotic resistance profiles of donor strains, recipient strains, and transconjugants, and the conjugation frequency of ESBL-encoding genes. Black squares denote the presence of resistance to a given antimicrobial agent.

Strain	Designation	Antimicrobial resistance										Conjugation frequency	ESBL-encoding gene
		AMP	AMC	CTX	CAZ	GEN	TET	CIP	NAL	SXT	CHL		
71	Donor	■		■	■		■		■	■			*bla*$_{CTX\text{-}M\text{-}55}$/*bla*$_{TEM\text{-}1}$
20	Recipient	■	■	■		■	■		■	■	■		
71-20	Transconjugant	■	■	■	■		■		■	■		6.2×10^{-1}	*bla*$_{CTX\text{-}M\text{-}55}$/*bla*$_{TEM\text{-}1}$
1-22	Recipient	■					■	■	■	■	■		
71-1-22	Transconjugant	■		■	■		■		■	■	■	3.3×10^{-4}	*bla*$_{CTX\text{-}M\text{-}55}$/*bla*$_{TEM\text{-}1}$
C600	Recipient								■				
71-C600	Transconjugant	■		■	■				■			1.5×10^{-2}	*bla*$_{CTX\text{-}M\text{-}55}$/*bla*$_{TEM\text{-}1}$
86	Donor	■		■	■		■		■	■	■		
86-20	Transconjugant	■	■	■	■		■		■	■	■	4.6×10^{-2}	*bla*$_{CTX\text{-}M\text{-}55}$
86-1-22	Transconjugant	■		■			■		■	■	■	1.2×10^{-2}	*bla*$_{CTX\text{-}M\text{-}55}$
86-C600	Transconjugant	■		■	■				■	■	■	4.2×10^{-2}	*bla*$_{CTX\text{-}M\text{-}55}$
122	Donor	■		■	■				■	■			
122-20	Transconjugant	■	■	■	■		■		■	■		9.1×10^{-2}	*bla*$_{CTX\text{-}M\text{-}55}$
122-1-22	Transconjugant	■		■			■		■	■	■	1.1×10^{-4}	*bla*$_{CTX\text{-}M\text{-}55}$
122-C600	Transconjugant	■		■	■				■			1.9×10^{-2}	*bla*$_{CTX\text{-}M\text{-}55}$

AMP: Ampicillin; AMC: Amoxicillin-Clavulanic acid; CTX: Cefotaxime; CAZ: Ceftazidime; GEN : Gentamicin; TET: Tetracycline;

CIP: Ciprofloxacin; NAL : Nalidixic acid; SXT: Sulfamethoxazole/Trimethoprim; CHL: Chloramphenicol.

References

1. Kiratisin, P.; Apisarnthanarak, A.; Laesripa, C.; Saifon, P. Molecular characterization and epidemiology of extended-spectrum-beta-lactamase-producing Escherichia coli and Klebsiella pneumoniae isolates causing health care-associated infection in Thailand, where the CTX-M family is endemic. *Antimicrob Agents Chemother* **2008**, *52*, 2818-2824, doi:10.1128/AAC.00171-08.
2. Archambault, M.; Petrov, P.; Hendriksen, R.S.; Asseva, G.; Bangtrakulnonth, A.; Hasman, H.; Aarestrup, F.M. Molecular characterization and occurrence of extended-spectrum beta-lactamase resistance genes among Salmonella enterica serovar Corvallis from Thailand, Bulgaria, and Denmark. *Microb Drug Resist* **2006**, *12*, 192-198, doi:10.1089/mdr.2006.12.192.
3. Qiao, J.; Alali, W.Q.; Liu, J.; Wang, Y.; Chen, S.; Cui, S.; Yang, B. Prevalence of Virulence Genes in Extended-Spectrum beta-lactamases (ESBLs)-Producing Salmonella in Retail Raw Chicken in China. *J Food Sci* **2018**, *83*, 1048-1052, doi:10.1111/1750-3841.14111.
4. Usha, G.; Chunderika, M.; Prashini, M.; Willem, S.A.; Yusuf, E.S. Characterization of extended-spectrum beta-lactamases in Salmonella spp. at a tertiary hospital in Durban, South Africa. *Diagn Microbiol Infect Dis* **2008**, *62*, 86-91, doi:10.1016/j.diagmicrobio.2008.04.014.
